# Supplementary material for: Quantitative Autofluorescence in Non-Neovascular Age Related Macular Degeneration
Source: Biomedicines. 2023 Feb 15;11(2):560. doi: 10.3390/biomedicines11020560 (PMC9952913; doi:10.3390/biomedicines11020560)
Supplement: Supplementary file 1 [file biomedicines-11-00560-s001.zip › biomedicines-2218437-supplementary.pdf]

Supplementary file

Contents

Figure S1: Flowchart showing the analysed participant cohort 2

Figure S2: Correlation between Age and qAF8 levels in AMD and age-matched controls. 3

Table S1. Participant and imaging characteristics in AMD categories based on the Beckman and OCT classification 4

Table S2: Age, gender and lens status adjusted associations between stage of SDD, qAF8 levels and drusen volume on risk factors of RPE cell death in AMD eyes 6

Figure S1: Flowchart showing the analysed participant cohort

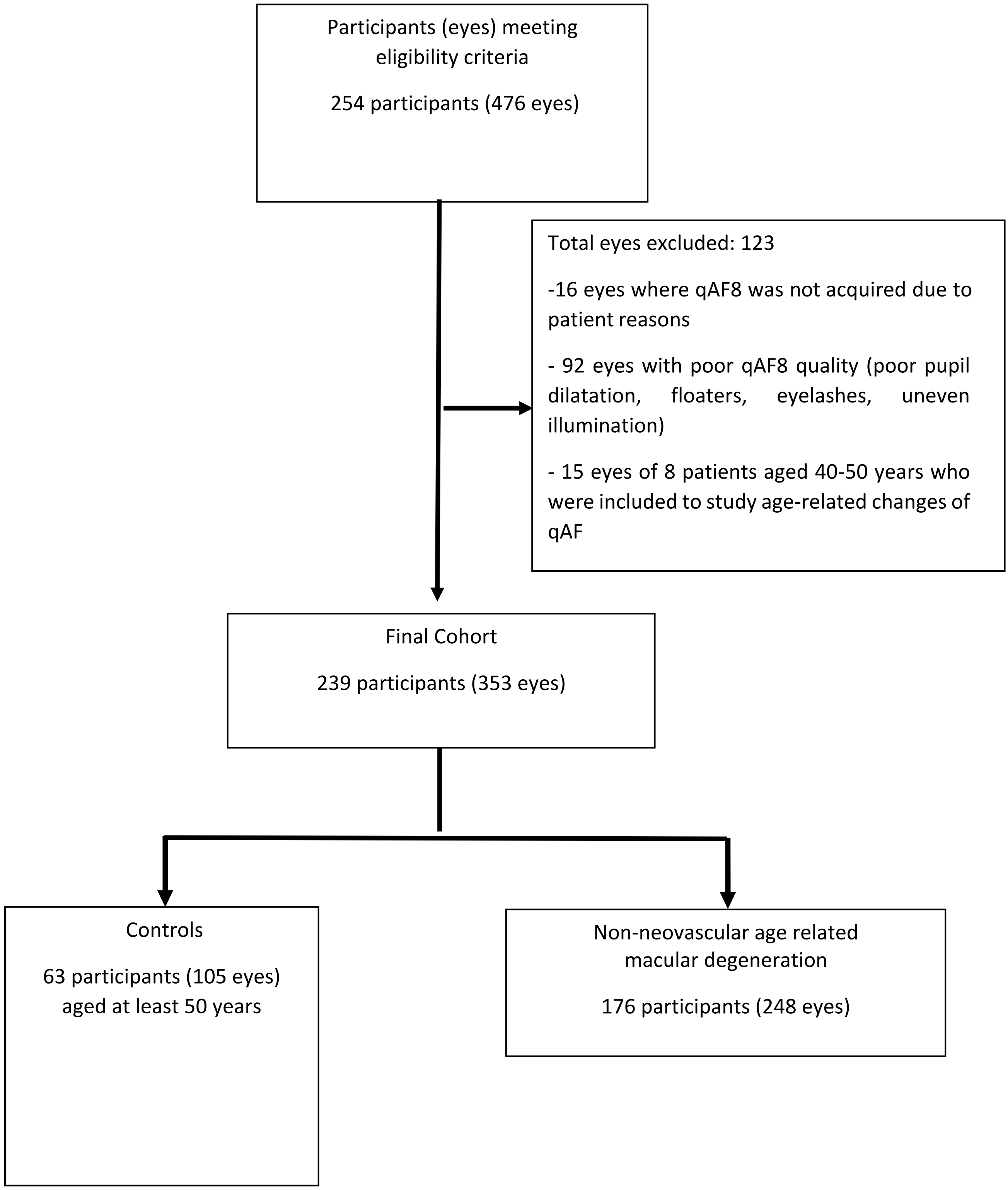

Figure S2: Correlation between Age and qAF8 levels in AMD and age-matched controls.

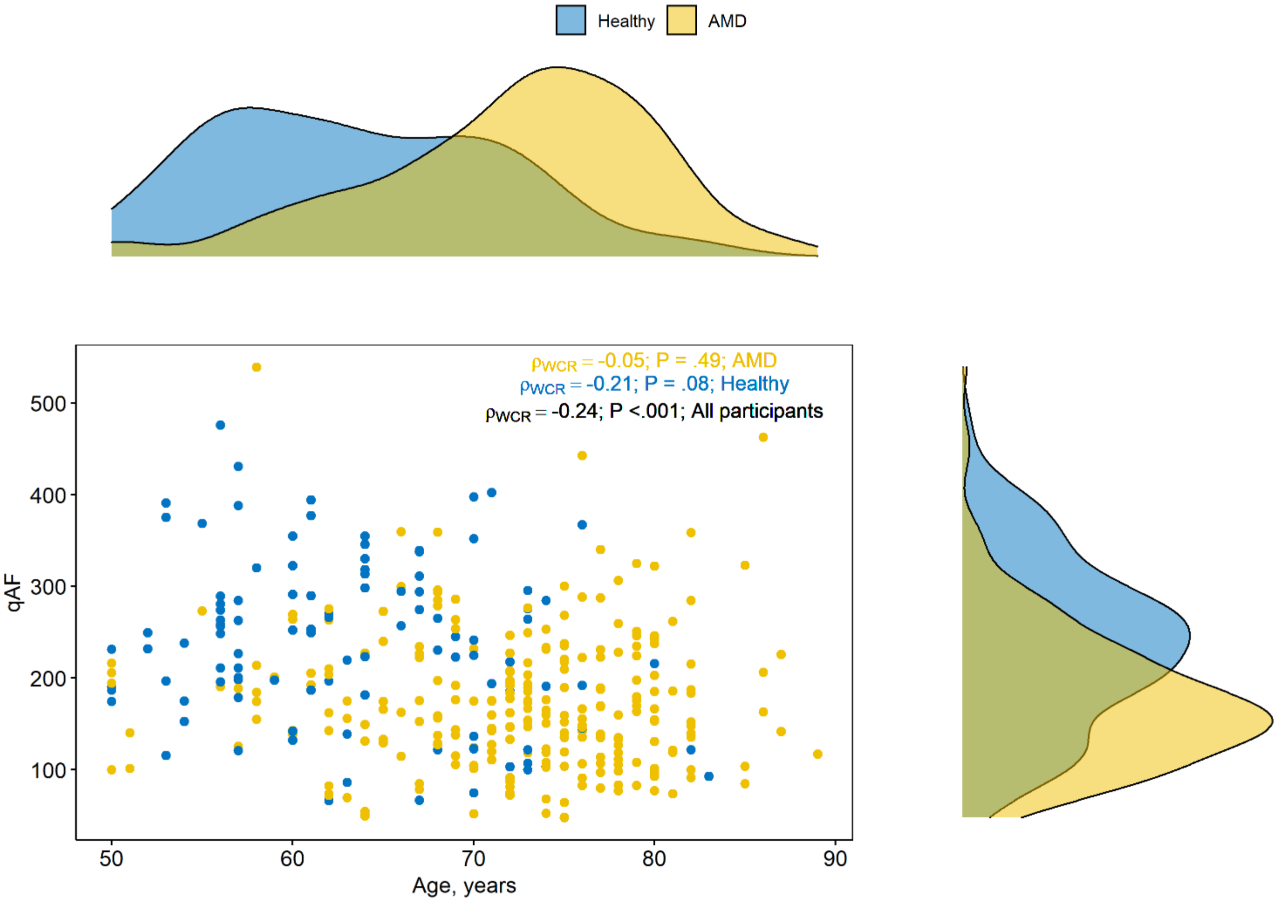

The two plots show the age distribution of the study participants (top) and qAF8 distribution on the right by AMD and healthy controls from participants aged 50 years or above. Abbreviations: WCR = within-cluster resampling

Table S1. Participant and imaging characteristics in AMD categories based on the Beckman and OCT classification

	Beckman AMD classification			AMD classification based on multimodal imaging			
**Variable**	Early, N=59 eyes of 50 participants	iAMD, N=161 eyes of 125 participants	GA, N=28 eyes of 22 participants	Early, N = 47 of 38 participants	iAMD, N = 76 of 58 participants	SDD, N = 97 of 75 participants	cRORA, N = 28 of 22 participants
**Participant level[a]**							
**Number of bilateral participants**	9 (18.0%)	36 (28.8%)	6 (27.3%)	9(23.7%)	18(31.0%)	22(29.3%)	6(27.3%)
**Age, years**	69.5 (8.5)	73.1 (7.1)	74.0 (5.1)	69.0 (8.2)	71.5 (7.0)	73.8(7.6)	74.0 (5.1)
**Age, years**							
< 60	5 (10.0%)	5 (4.0%)	1 (4.6%)	4 (10.5%)	4(6.9%)	3(4.0%)	1(4.6%)
60-69	21 (42.0%)	30 (24.0%)	1 (4.6%)	17 (44.7%)	17(29.3%)	14(18.7%)	1(4.6%)
70-79	17 (34.0%)	68 (54.4%)	17 (77.3%)	14 (36.8%)	30(51.7%)	41(54.7%)	17(77.3%)
>=80	7 (14.0%)	22 (17.6%)	3 (13.6%)	3 (7.9%)	7(12.1%)	17(22.7%)	3(13.6%)
**Gender**							
M	21 (42.0%)	42 (33.6%)	11 (50.0%)	18(47.4%)	20(34.5%)	24(32.0%)	11(50.0%)
F	29 (58.0%)	83 (66.4%)	11 (50.0%)	20(52.6%)	38(65.5%)	51(68.0%)	11(50.0%)
**Eye level[b]**							
**Pseudophakia**	12 (20.3%)	35 (21.7%)	6 (21.4%)	9 (19.1%)	16 (21.1%)	22 (22.7%)	6 (21.4%)

**Median qAF8**	201.1 (141.1, 233.1)	154.7 (119.7, 206.3)	159.9 (115.5, 185.5)	213.8 (157.7, 258.3)	149.0 (118.3, 185.0)	156.4 (119.1, 217.1)	159.9 (115.5, 185.5)
**Stage of SDD**							
No SDD	41 (69.5%)	82 (50.9%)	28 (100.0%)	47 (100.0%)	76 (100.0%)	0 (0%)	28 (100.0%)
Stage 1	1(1.7%)	4 (2.5%)	0 (0.0%)	0 (0%)	0 (0%)	5 (5.2%)	0 (0%)
Stage 2	1 (1.7%)	14 (8.7%)	0 (0.0%)	0 (0%)	0 (0%)	15 (15.5%)	0 (0%)
Stage 3	16 (27.1%)	61 (37.9%)	0 (0.0%)	0 (0%)	0 (0%)	77 (79.4%)	0 (0%)
**Drusen volume in those without cRORA**	-	-	-	0.3 (0.3, 0.4)	0.5 (0.4, 0.5)	0.5 (0.4, 0.6)	NA
**Stages of RPE cell integrity**							
Absence of HRF without evidence of iRORA or cRORA	-	-	-	46 (97.9%)	54 (71.1%)	53 (54.6%)	0 (0%)
Presence of HRF without evidence of iRORA or cRORA	-	-	-	1 (2.1%)	8 (10.5%)	7 (7.2%)	0 (0%)
Presence of iRORA without any evidence of cRORA	-	-	-	0 (0%)	14 (18.4%)	37 (38.1%)	0 (0%)
Presence of cRORA	-	-	-	0 (0%)	0 (0%)	0 (0%)	28 (100.0%)

This table includes controls, aged 50 years or older and AMD eyes categorised based on the Beckman Classification on colour fundus photographs. We also include the multimodal based classification to highlight the incorporation of SDD and other parameters to each category.

[a] Participant level characteristics were based on total cohort of 231 participants, the number of participants across Beckmann categories do not add up to 231 (total number of participants in the cohort) due to bilateral eligibility in the cohort where eyes from the same participant belong in different Beckmann classification groups.

[b] Eye level characteristics based on total analysed sample of 353 eyes.

Abbreviations: AMD – age related macular degeneration; cRORA – complete retinal pigment epithelium and outer retinal atrophy; GA – Geographic atrophy; HRF – Hyperreflective foci; iAMD – intermediate AMD; IR- Infrared reflectance; iRORA – incomplete retinal pigment epithelium and outer retinal atrophy; IQR – Interquartile range; OCT – Optical Coherence Tomography; qAF8 – quantitative autofluorescence; RPE – Retinal pigment epithelium; SDD – subretinal drusenoid deposits

Table S2: Age, gender and lens status adjusted associations between stage of SDD, qAF8 levels and drusen volume on risk factors of RPE cell death in AMD eyes

AMD eyes only				
Variable	Absence of HRF or iRORA or cRORA, N = 153 eyes [total No] of 117 participants	Presence of HRF, iRORA or cRORA, N = 95 eyes [total No] of 70 participants	Adjusted odds ratio (95% CI)[a]	P-value
**Stage of SDD**				
stage 0	100 (65%)	51 (54%)	Ref	-
stage 1 or 2	11 (7.2%)	9 (9.5%)	0.27 (0.03-2.16)	.22
stage 3	42 (27%)	35 (37%)	1.44(0.87-2.38)	.16
**Median qAF8, (IQR) or per 10% increase in qAF8**	175 (135, 231)	152 (117, 188)	0.91(0.85-0.97)	**.006**
**Drusen volume in those without cRORA, median (IQR) or per 0.1 unit increase**	0.40 (0.34, 0.47) [153]	0.54 (0.46, 0.68) [67]	2.00(1.47-2.73)	**<.001**

[a] Adjusted for age, gender and lens status

Abbreviations: AMD – age related macular degeneration; CI – Confidence Interval; cRORA – complete retinal pigment epithelium and outer retinal atrophy; GA – Geographic atrophy; HRF – Hyperreflective foci; iRORA – incomplete retinal pigment epithelium and outer retinal atrophy; IQR – Interquartile range; qAF8 – quantitative autofluorescence; RPE – Retinal pigment epithelium.
